# Estimation of interface frictional anisotropy between sand and snakeskin-inspired surfaces

**DOI:** 10.1038/s41598-023-31047-3

**Published:** 2023-03-09

**Authors:** Seung-Hun Lee, Muhammad Naqeeb Nawaz, Song-Hun Chong

**Affiliations:** Department of Civil Engineering, Sunchon National University, 255, Jungang-Ro, Sunchon-Si, Jeollanam-Do 57922 Republic of Korea

**Keywords:** Civil engineering, Structural materials

## Abstract

The transmission of loads across the soil-structure mobilizes direction-dependent shear resistance, which can be selectively used to design geo-structures. A previous study confirmed the frictional anisotropy induced by the interface between the soil and snakeskin-inspired surfaces. However, it is necessary to estimate the interface friction angle quantitatively. In this study, a conventional direct shear apparatus is modified, and 45 cases are performed in two-way shearing directions between bio-inspired surfaces and Jumunjin standard sand under three vertical stresses (50, 100, and 200 kPa). The results show that: (1) shearing against the scales (cranial shearing) mobilizes larger shear resistance and produces a dilative response than shearing along the scales (caudal shearing) and (2) higher scale height or shorter scale length exhibits dilative tendency and produces higher interface friction angle. Further analysis is conducted to capture the frictional anisotropy as a function of the scale geometry ratio, which reveals that the interface anisotropy response is more pronounced during cranial shearing in all the cases, and the difference in the interface friction angle for the caudal → cranial test is higher than that for the cranial → caudal test at the given scale ratio.

## Introduction

The concept of bio-inspired geotechnics for solving geotechnical engineering problems has gained significant attention over the past few years^1–6^. The development of creative design of geotechnical systems have been influenced by what nature has done over the billions of years^4,7–11^. The problem-driven approach in bio-geotechnics includes identifying the geotechnical problem, utilizing biological analogy, and ultimately devising an efficient solution for design practice.

Load transfer across the soil-structure interface plays a crucial role in securing the load-carrying capacity and improving the efficiency of geotechnical engineering application. For example, axially loaded piles require a large shear resistance to increase the load-carrying capacity, whereas the pile-driving process minimizes the mobilized shear resistance. The selection of the loading direction significantly affects the capacity and execution of geo-structures.

Several studies have shown that snakes use the ventral scales underneath their body while performing forward and backward movement^8,12–16^. The movement of a snake against its ventral scales is referred to as cranial movement, whereas the movement along the scale is known as caudal movement^8,13,17^. Snakeskin has proved to be effective in utilizing the frictional anisotropy across the soil-structure interface. The characteristic behavior of the friction pile during the installation and pullout activity of jacking was explored using the asymmetric surfaces inspired by ventral scales of snakes for analysis. It was observed that the pile under a pull-out load requires a higher friction resistance compared to that during the installation of jacking^18^. Furthermore, the direct shear tests with a snakeskin-inspired plate were conducted, which showed that the interface shear response when sheared in the cranial direction (against the ventral scales) was similar to that of the soil interface with rough surfaces; conversely, caudal shearing showed a response similar to that of soil with smooth surfaces^19^.

The frictional anisotropy induced by snakeskin-inspired surfaces was studied using interface direct shear tests on two different sands under a vertical stress of 75 kPa. The results showed that the cranial shearing direction mobilizes a larger peak and residual interface strength and dilation compared to that of the caudal shearing direction. Particle image velocimetry analysis revealed larger soil deformations and dilation induced within the soil during cranial shearing. Shearing in the cranial direction enables soil to latch on scales and increases the contact area and contact soil behind the scales. Eventually, wedges tend to develop at the leading front of the scales where soil displacement, shear strains, and volumetric strains are localized. The soil within the wedges developed during shearing experienced a local increase in the mean effective stresses. However, the caudal shearing direction hinders the latching of the soil on the scales and reduces the contact area^8^. However, there is the need to estimate the interface friction angle quantitatively with different snake-inspired surfaces and two-way shearing directions under different loads.

The present study explores the interface shear response with the aid of a modified direct shear apparatus by extending a previous study by the authors (Martinez et al.^8^) to quantify the shear stress–strain response and the corresponding interface friction angle under three different vertical stresses. The conventional direct shear apparatus is modified to ensure the constant application of vertical stress during the shearing phase and to accommodate bio-inspired surfaces inside the lower shear box. To evaluate the interface frictional angle, two-way direct shear tests are performed using a modified direct shear apparatus. The results are analyzed to obtain a better understanding of how scale geometry affects the interface shear response and frictional anisotropy. The discussion summarizes the interface friction angle as a function of the scale geometry ratio to characterize the interface load transfer mechanism and the potential adoption of snake-inspired surfaces in engineering applications.

## Modification of direct shear test

**Figure 1:**
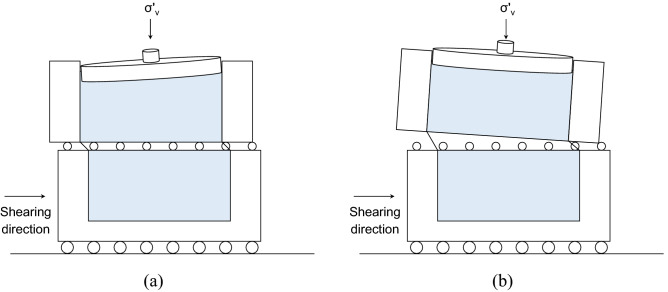
Common limitations of conventional direct shear apparatus: (**a**) tilting of loading plate; (**b**) rotation of upper shear box (modified from Jewell and Wroth^21^).

**Figure 2:**
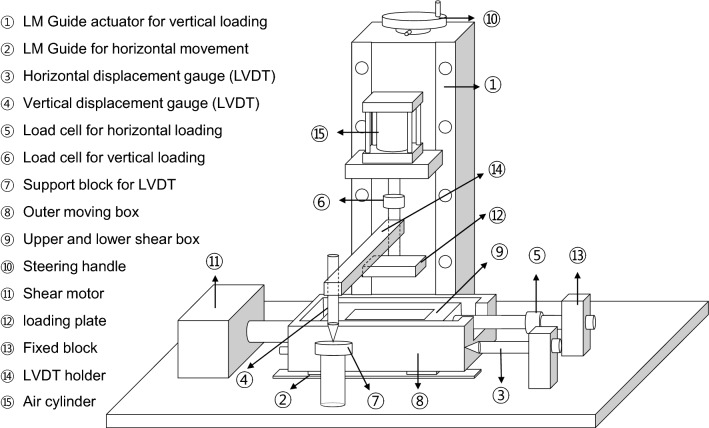
Schematic of direct shear apparatus invented in this study.

Direct shear test has been widely used to define the shear strength parameters based on the Mohr–Coulomb failure criterion. However, the conventional direct shear test apparatus causes an unfavorable shear response, which hinders the accurate evaluation of shear strength parameters^20–23^. In a direct shear system, a constant vertical stress is applied to the top loading plate to achieve dilative or contractive soil deformation. Figure 1 shows two common limitations: (1) tilting of the loading plate and (2) rotation of the upper shear box, which eventually results in unstable vertical loading during the shearing process^20,21^. This study modifies the shear boxes to overcome these potential problems and accommodate bio-inspired surfaces inside the lower shear box (Fig. 2). The vertical stress application through the loading rod mounted on a steel ball and rectangular loading plate in the conventional apparatus is replaced by a rectangular loading plate attached to a vertical loading frame. Note that loading plate and loading rod were not fixed in the conventional apparatus. The length, width, and thickness of the rectangular steel plate are 100, 63, and 20 mm, respectively. In addition, a linear motion guide (LM guide) is installed for smooth and frictionless movement of the lower shear box bolted to an outer moving box. The lower shear box is a platform over which the bio-inspired surface is fastened to perform interface tests. The upper and lower parts of the shear box are detachable and are bolted before the test. The length, width, and thickness of the upper shear box are 101, 63.5, and 24 mm, whereas those of the lower part of the shear box are 162, 120, and 25 mm, respectively. The direct shear system includes an air cylinder that applies a vertical load to the specimen, a shear motor that applies a shear force, and an integrated data acquisition system. The vertical and horizontal displacements are measured with LVDTs (linear variable displacement transducers). The vertical and horizontal loads applied to the sand specimens are measured using the load cells. A reaction arm that connects the fixed block to the upper shear box transfers the friction force generated at the soil–textured plate interface to the horizontal load cell.

## Experimental materials and methods

### Bio-inspired surfaces

**Figure 3:**
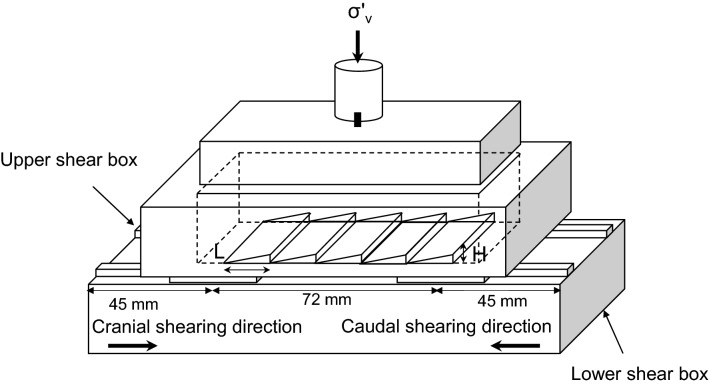
Geometry of bio-inspired surface and two-way shearing directions. The surface has 72 mm textured part at center and 45 mm untextured part at both sides. Cranial shearing direction is that soil moves against scales. In contrast, soil moves along the scales in the caudal shearing direction. The LM guide is installed between the upper shear box and lower shear box, and then facilitates moving of the lower shear box.

In this study, seven bio-inspired surfaces made of polycarbonate material are tested. The details and idealization of the bio-inspired surfaces can be found in previous studies^8,19^. Each surface consists of 72 mm textured central part and 45 mm untextured part on both sides to minimize the boundary effect originating from the sidewalls of the shear box (Fig. 3). The different combinations of scale geometries are summarized in Table 1. Note that the total length of the surface scales is fixed as 72 mm in all plates.

**Table 1:** Geometrical parameters of bio-inspired surfaces. N indicates the number of surface scales. Total length of surface scales is fixed as 72 mm in all plates (details refer to Fig. 3). The normalized roughness R_n_ is used to describe the effect of surface roughness on the interface behavior that can be obtained by R_n_ = H/D_50_^24^. Where H is the maximum scale height (mm), and D_50_ is the average particle size (mm). Normalized roughness of an untextured surface is close to zero.

No	Type	Scale length L [mm]	Scale height H [mm]	Normalized roughness R_n_ [mm]	N [ ]
1	Textured	6	0.3	0.520	12
2		12	0.1	0.175	6
3		12	0.3	0.520	6
4		12	0.45	0.789	6
5		12	0.72	1.263	6
6		18	0.3	0.520	4
7		24	0.3	0.520	3
8	Untextured	–	–	Close to 0	–

### Sand specimen

Jumunjin standard sand is used; and its basic properties are listed in Table 2. To achieve a relative density of 40% by varying the specimen height from 22 to 24 mm, all the specimens are air-pluviated over the bio-inspired surface in the shear box.

**Table 2:** Basic properties of Jumunjin standard sand used in this study. Friction angle is determined using circular shear box of 60 mm diameter.

Properties	Value
Coefficient of curvature C_c_ [ ]	0.920
Uniformity coefficient C_u_ [ ]	1.480
Average particle size D_50_ [mm]	0.57
Maximum void ratio e_max_ [ ]	0.919
Minimum void ratio e_min_ [ ]	0.625
Specific Gravity G_s_ [ ]	2.621
Friction angle φ_peak_ [°]	37.0
Friction angle φ_residual_ [°]	36.0

### Interface direct shear tests

The sand specimens are sheared until a displacement of 10 mm (10% shear strain) failure criteria at a shear rate of 1 mm/min. As shown in Fig. 3, two-way shearing directions are applied and composed of (1) cranial direction during the first half cycle and then caudal shearing during the second half cycle (cranial → caudal test) and (2) caudal direction during the first half cycle and cranial shearing during the second half cycle (caudal → cranial test). All the sensors are connected to the data logger to record and save data, automatically while the LabView program is used for continuous monitoring. The shear stress mobilized at the interface is calculated by dividing the measured shear force by the cross-sectional area of the specimen.

## Results and analysis

A total of 45 interface direct shear tests are performed on seven bio-inspired surfaces and one untextured surface under three initial vertical stresses (50, 100, and 200 kPa) and two-way shearing directions. The interface friction angle with a varying scale geometry is quantified by selecting the peak shear stress at the corresponding vertical stress and shearing direction based on the Mohr Coulomb theory. The correlation coefficient R^2^ is found out to be 0.99 in all the cases. All the data sets, including the interface friction angle, are summarized in Table. 3.

**Table 3:** Summary of interface friction strength using modified direct shear apparatus with various scale geometries, three vertical stresses, and two-way shearing directions: (a) Cranial → Caudal; (b) Caudal → Cranial.

L [mm]	H [mm]	(a) Cranial → Caudal					
		Cranial first shearing			Caudal second shearing		
		σ′_v_ [kPa]	τ_peak_ [kPa]	ϕ_peak_ [°]	σ′_v_ [kPa]	τ_peak_ [kPa]	ϕ_peak_ [°]
6	0.3	50	60.9	45.1	50	24.3	28.7
		100	109.5		100	49.5	
		200	193.5		200	112.9	
12	0.3	50	48.2	41.4	50	19.7	23.9
		100	96.3		100	37.8	
		200	171.7		200	72.4	
18	0.3	50	40.9	32.3	50	18.3	20.5
		100	84.1		100	40.1	
		200	113.3		200	73.5	
24	0.3	50	30.9	28.4	50	17.9	20.6
		100	57.5		100	36.7	
		200	105.4		200	76.0	
12	0.1	50	23.7	24.1	50	16.9	21.4
		100	43.7		100	34.6	
		200	89.7		200	81.5	
12	0.45	50	64.3	45.1	50	18.2	26.9
		100	108.7		100	47.0	
		200	193.0		200	105.6	
12	0.72	50	64.8	46.6	50	25.9	32.2
		100	115.1		100	65.2	
		200	203.6		200	126.3	

L [mm]	H [mm]	(b) Caudal → Cranial					
		Caudal first shearing			Cranial second shearing		
		σ′_v_ [kPa]	τ_peak_ [kPa]	ϕ_peak_ [°]	σ′_v_ [kPa]	τ_peak_ [kPa]	ϕ_peak_ [°]
6	0.3	50	24.8	25.3	50	65.4	45.7
		100	50.3		100	114.1	
		200	92.9		200	196.4	
12	0.3	50	17.8	21.1	50	58.5	43.0
		100	40.9		100	103.4	
		200	76.3		200	178.6	
18	0.3	50	21.2	20.8	50	44.9	37.3
		100	39.8		100	91.7	
		200	74.6		200	143.2	
24	0.3	50	21.2	20.6	50	36.9	30.9
		100	38.9		100	68.6	
		200	74.0		200	114.0	
12	0.1	50	17.0	17.7	50	32.9	30.7
		100	26.9		100	48.8	
		200	66.3		200	123.5	
12	0.45	50	23.4	23.6	50	65.5	45.3
		100	43.4		100	109.5	
		200	81.6		200	194.3	
12	0.72	50	22.7	32.1	50	66.5	45.8
		100	66.9		100	115.9	
		200	125.6		200	195.2	
Untextured		50	12.1	13.9	50	12.1	13.9
		100	27.7		100	27.7	
		200	48.3		200	48.3	

### Interface shear behavior of bio-inspired surfaces

**Figure 4:**
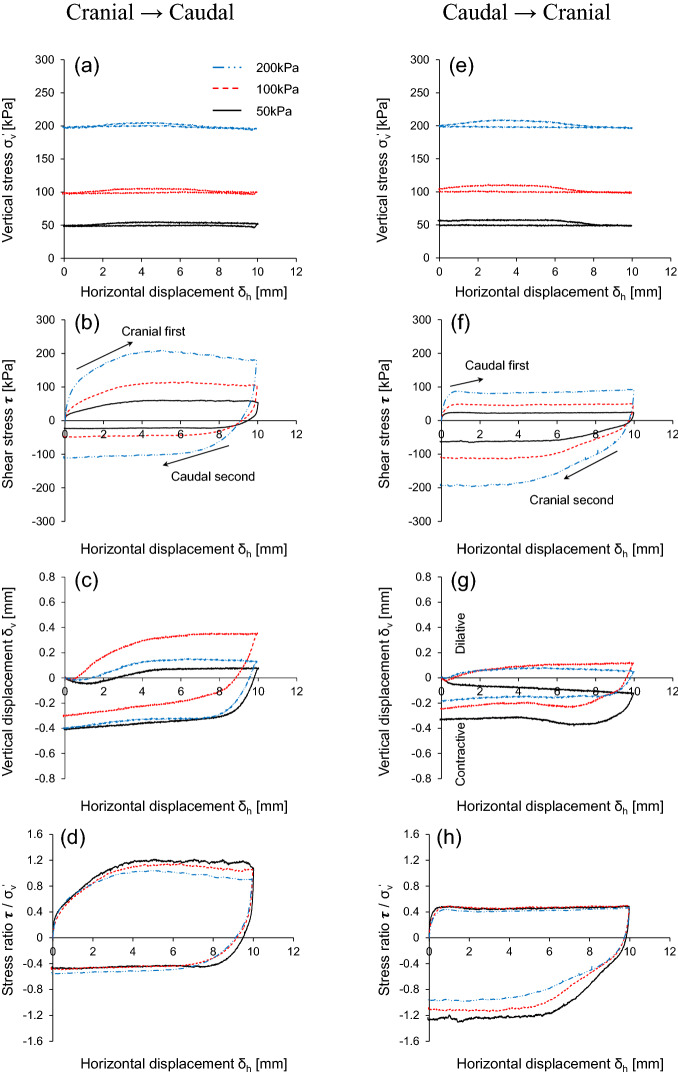
Response of interface frictional anisotropy using modified direct shear apparatus: Horizontal displacement against (**a** and **e**) Vertical stress; (**b** and **f**) Shear stress; (**c** and **g**) Vertical displacement; (**d** and **h**) Stress ratio. Experimental case is L = 6 mm and H = 0.3 mm.

Figure 4 shows the interface frictional anisotropy under three vertical stresses with the same scale geometry (L = 6 mm and H = 0.3 mm). The shear responses are plotted against the horizontal displacement related to the shear strain. As shown in Figs. 4(a) and (e), the modified direct shear apparatus invented in this study produces a nearly constant application of vertical stress during both the shearing sequences. There is a small fluctuation in the vertical stress during the cranial shearing direction, in which sand moves against the scales. This is because the passive zone between the scale and sand propagates toward the loading plate and eventually increases the local mean effective stress^8^. However, the application of vertical stress remains constant during caudal shearing owing to the absence of a passive zone along the scales. The constant vertical stress during the shearing process guarantees the accuracy of the modified direct shear apparatus. Figure 4(b) and (f) show that a higher vertical stress produces a larger shear stress, which is considerably higher for the cranial → caudal test as compared to that for the caudal → cranial test. While the cranial first shearing direction mobilizes higher shear resistance, the following caudal shearing reduces the shear stress. Conversely, caudal first shearing mobilizes the lower shear resistance and cranial second shearing enhances shear stress. Regardless of the starting direction, cranial shearing exhibits higher shear stress in all the cases. Previous study^8^ explored the effect of shearing direction and scale geometry on induced soil deformation by analyzing particle image velocimetry. Shearing in the cranial direction enables soil to latch on scales and increases the contact area and contact soil behind the scales. Eventually, wedges tend to develop at the leading front of the scales where the soil displacement, shear strains, and volumetric strains are localized. The soil within the wedges developed during shearing experiences a local increase in the mean effective stresses. However, the caudal shearing direction hinders latching of the soil on the scales and reduces the contact area.

The induced failure deformation is evenly distributed along the soil-scale interface; thus, the lack of well-defined wedges produces shear bands. The volumetric response, either contraction or dilation, is mainly affected by the geometry of the surface and vertical stress. In the vertical displacement, cranial shearing during the first cycle indicates a higher tendency to dilate, as shown in Fig. 4(c), than caudal shearing, as illustrated in Fig. 4(g). Shearing in the cranial direction causes dilation of soil at the leading front of scales, whereas caudal shearing contracts sand specimens at the tailing end. In the cranial → caudal shearing direction, the increase in vertical stress from 50 to 100 kPa causes a pronounced dilative tendency; however, it decreases at 200 kPa. During caudal first sharing (caudal → cranial), contractive behavior is observed at low vertical stress (50 kPa), while higher vertical stress (100 kPa and 200 kPa) shows a dilative tendency. Figure 4(d) and (h) show the stress ratio computed as the shear stress divided by the vertical stress applied to the specimen. As expected, cranial shearing mobilizes higher shear resistance than caudal shearing.

### Effect of scale geometry

**Figure 5:**
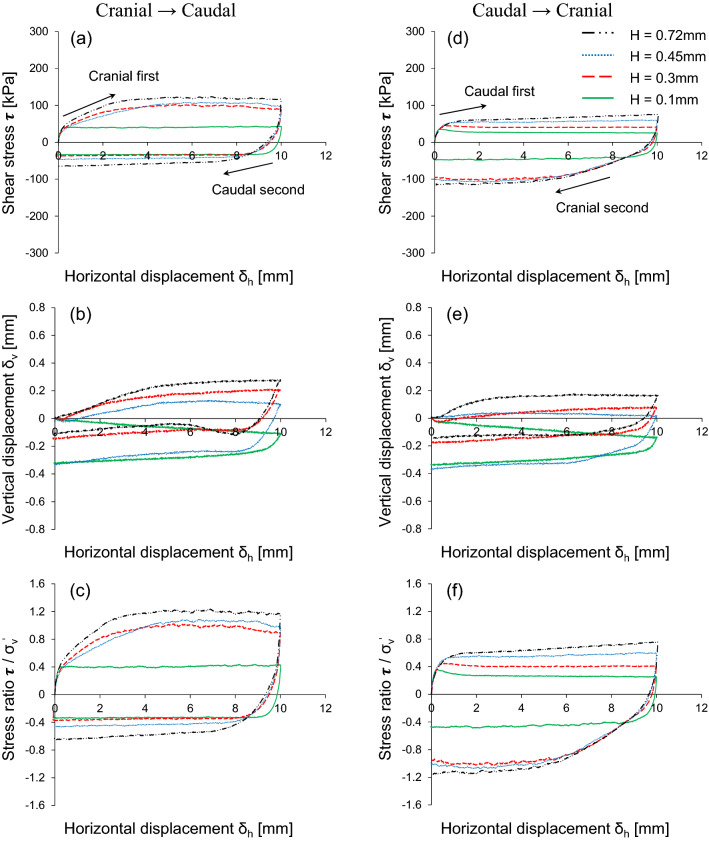
The effect of scale height on the interface shear response: Horizontal displacement against (**a** and **d**) Shear stress; (**b** and **e**) Vertical displacement; (**c** and **f**) Stress ratio. For all the cases, the applied vertical stress is 100 kPa and scale length L is fixed as 12 mm.

Figure 5 shows the results of the interface shear behavior by varying the scale height while keeping the scale length constant at 12 mm. A higher scale height mobilizes larger shear resistance in both the sequence of shearing (i.e., cranial → caudal and caudal → cranial tests), as shown in Figs. 5(a) and (d). In addition, cranial shearing direction produces larger shear stress. The scale height changes the tendency of the vertical displacement associated with the volumetric response, as presented in Figs. 5(b) and (e). The surface with a low scale height (H = 0.1 mm) exhibited contractive behavior, whereas responses at relatively higher scale heights (H > 0.1 mm) are dilative. This is because a higher height increases the interface roughness and develops larger individual passive wedges with dilation on the soil ahead of the scale and contraction on the soil behind the scale. The stress ratio increases with higher scale height during the cranial first and cranial second shearing directions. Conversely, a smaller scale height reduces the stress ratio, as shown in Figs. 5(c) and (f). The untextured scale with a smooth surface shows the same shear stress and vertical displacement with a low scale height in each shearing direction (not presented here).

**Figure 6:**
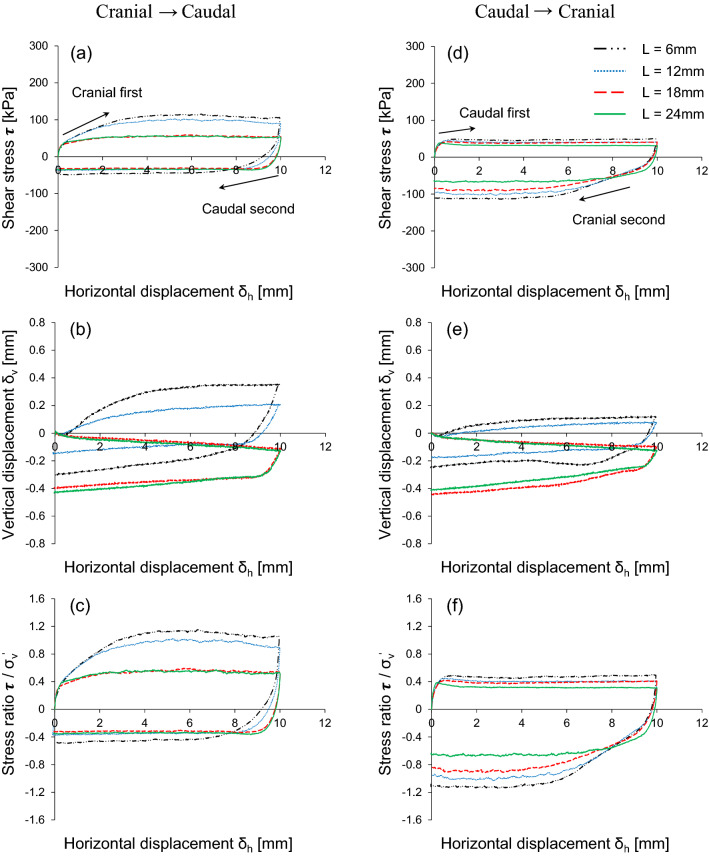
Effect of scale length on the interface shear response: Horizontal displacement against (**a** and **d**) Shear stress; (**b** and **e**) Vertical displacement; (**c** and **f**) Stress ratio. The applied vertical stress is 100 kPa and scale height H is fixed as 0.3 mm in all the cases.

Figure 6 shows the response of the interface shear behavior by varying the scale length. A shorter scale length mobilizes higher shear resistance during both the cranial → caudal and caudal → cranial tests, as shown in Figs. 6(a) and (d). A surface with shorter scale lengths has more scales and thus increases the shear stress. Note that the total length of the surface scales is fixed at 72 mm for all plates. The smaller scale lengths (L = 6 mm and L = 12 mm) exhibit a dilative tendency. This is attributed to the fact that more textured surfaces (i.e., shorter scale lengths) mobilize individual wedges and the overall volumetric response is dilative. In contrast, a relatively longer scale lengths (L = 18 mm and 24 mm) show a contractive response [Figs. 6(b) and (e)]. As expected, the stress ratio is higher for surfaces with smaller scale lengths than for surfaces with longer scale lengths, as shown in Figs. 6(c) and (f), during both shearing cycles. The untextured scale without any scale height behaves similarly to a longer scale length (L = 24 mm).

### Evolution of interface friction angle

**Figure 7:**
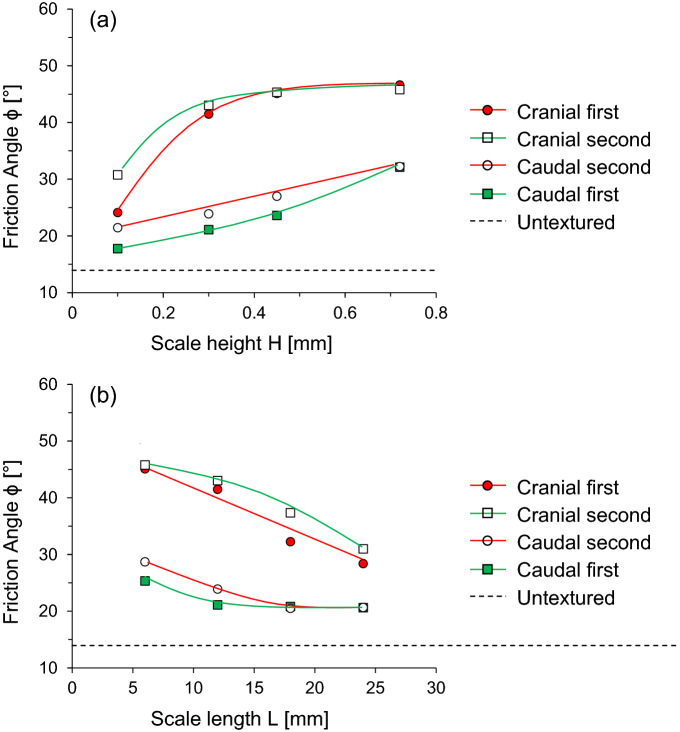
Interface friction angle as a function of snakeskin-inspired scale geometry and two-way shearing directions: (**a**) Scale height (L = 12 mm fixed); (**b**) Scale length (H = 0.3 mm fixed).

Figure 7(a) shows the evolution of the interface friction angle with varying scale heights at a constant scale length (L = 12 mm) in different shearing directions. The interface friction angle is observed to increases with an increase in the scale height, and cranial shearing produces a higher interface friction angle than caudal shearing. However, this increasing tendency is affected by the shearing direction. For the cranial first case, the difference between the friction angle at 0.1 mm and 0.72 mm scale height is 22.5°. For the cranial second case, it is 15.1° at the given scale length. There is a sharp increase in interface friction angle against surfaces with scale height between 0.1 and 0.3 mm during cranial shearing. Subsequently, an increase in the height results in a slight increase in the interface friction angle. A previous study analyzed the effect of surface roughness on sand-steel interface behavior^25^. In this study, the increase in the surface roughness related to the scale height produced a higher interface frictional strength, yet it reaches an asymptotic value at a certain scale height. Meanwhile, caudal shearing, exhibits a linear increase in the interface friction angle, regardless of its sequence. Furthermore, the difference in the interface angle is relatively moderate compared to cranial shearing. The difference in friction angle at 0.1 mm and 0.72 mm scale height are observed to be 14.4° and 10.8° for caudal first and second cases, respectively.

Figure 7(b) shows the evolution of the interface friction angle with varying scale lengths at constant scale height (H = 0.3 mm) in different shearing directions. During cranial shearing, the interface friction angle dramatically decreases with the increasing scale length. At the given scale height, the difference between the friction angle at the 6 and 24 mm scale lengths are 17° and 15° for cranial first and second cases, respectively. Similarly, the difference in the interface angles is 5° for the caudal first case and 8° for the caudal second case. However, compared with cranial shearing, the difference is insignificant. Regardless of the scale geometry, the second shearing in either the cranial or caudal direction produces a higher friction angle than the first shearing up to a certain limit and becomes nearly constant afterwards. The first shearing process propagates the interface failure zone initiated from sand particles at the trailing end of scales (space among scales) and increases the mean effective stress. Subsequently, more compacted sands around the scales produce higher shear resistance during the second shearing cycle. Correspondingly, this mechanism enhances the interface friction angle during the second cycle in both cases of the shearing sequence. Note that the interface friction angle from untextured surface shows the lowest interface friction angle.

## Discussion and implications to geotechnical application

**Figure 8:**
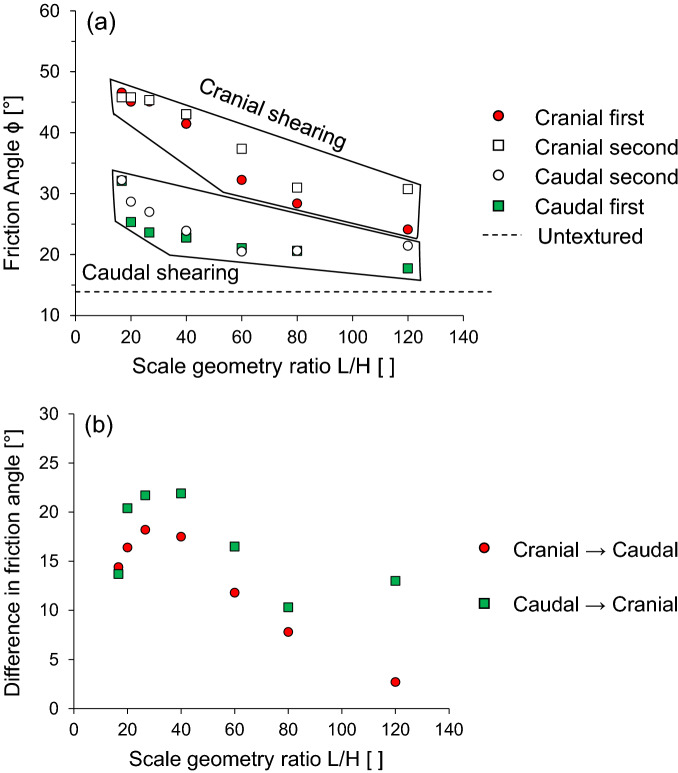
Change in the interface friction angle as a function of normalized scale geometry L/H ratio: (**a**) Interface friction angle for cranial and caudal shearing directions; (**b**) Difference of interface friction angle for both the shear directions (cranial → caudal and caudal → cranial).

In the previous sections, the interface shear response is explored through parametric tests, including various scale geometries inspired by snakeskin, initial vertical stress, and two-way shearing directions. Furthermore, the scale geometry ratio, which is defined as the ratio of scale length to height, is used to further analyze the interface frictional anisotropy. Figure 8(a) shows the interface friction angle as a function of L/H ratio. Compared to caudal shearing, cranial shearing produces a higher interface friction angle at all scale geometry ratios, regardless of shearing sequence. The interface friction angle between the cranial and caudal shearing directions varies from 2.7° to 17.8° for cranial → caudal and 10.3° to 21.9° for caudal → cranial. The interface friction angle significantly decreases as the L/H ratio increased from 16.67 to 80, and gradually decreases between L/H = 80 to L/H = 120 in the cranial and caudal shearing directions. Note that a large L/H can be indicated as a lower scale height at the same scale length. The scales of the surfaces with a large L/H form shear bands with more uniform soil deformation, while a small L/H mobilizes the interface soil resistance developed by a well-defined passive wedge ^8^. Figure 8(b) presents the difference in the interface friction angle that occurs during each shearing process. At a scale geometry ratio L/H = 16.6, the difference in the interface friction angle is 14.4° for cranial → caudal and 13.7° for caudal → cranial. The difference in the interface friction angle between cranial → caudal and caudal → cranial is 0.7°. In the other cases, where L/H varies from 20 to 80 for both the shearing directions, the difference in the interface friction angle is approximately 4°. However, the absolute difference in the interface friction angle is 10.3° (2.7° for cranial → caudal and 13° for caudal → cranial) with L/H = 120. Thus, difference in the interface friction angle depends on the shearing direction. A test case with cranial → caudal shows a less difference than that of caudal → cranial test case. This is because caudal first shearing process densifies the sand around the scale and following cranial second shearing direction mobilizes higher interface shear resistance against the denser sand. However, cranial first shearing develops a larger individual passive wedge, and the opposite shearing direction (caudal second) follows the uniform shear bands. Regardless of the shearing direction, difference in the interface friction angle increases with the increase in scale geometry ratio up to a certain limit and then decreases afterwards.

The interface friction angle obtained from various scale geometries and different shearing directions helps to understand the interface shear response, and ultimately bioinspired geo-structures (e.g., driven piles, offshore monopiles, soil anchors, and tunnel boring machines) can efficiently select the interface friction angle. For example, a driven-pile inspired by a relatively higher L/H scale ratio minimizes the mobilized shear resistance. The outcome of this study is not limited to bio-inspired surfaces but is also applicable to other geotechnical infrastructure related interface shearing systems such as textured geomembranes and ribbed soil reinforcements.

## Conclusions

This study uses a modified direct shear test apparatus for two-way shearing directions (i.e., cranial → caudal and caudal → cranial tests) on Jumunjin standard sand sheared against bio-inspired surfaces to quantify interface frictional anisotropy. The salient conclusions are as follows:

- The proposed modifications in the shear boxes and vertical loading assembly result in the constant application of vertical stress by preventing the irregular distribution of stresses in soils during the shearing process, and thus eventually enhance the accuracy in determining the interface friction angle.
- Shearing against the scales (cranial shearing) mobilizes a larger shear resistance with a dilative tendency, while shearing along the scales (caudal shearing) induces a lower shear resistance with a contractive response.
- At a given scale geometry, either height or length, higher scale height or shorter scale length mobilizes a larger shear resistance in both the shearing sequences and exhibits a dilative tendency. A higher scale height indicates a rough surface that induces passive wedges around the scales to increase the shear resistance. Meanwhile, shorter scales correspond to a greater number of scales that produce a higher shear resistance and dilation.
- A larger height and shorter scale length produces a higher interface friction angle regardless of the shearing directions. The interface friction angle shows a dramatic increase during the cranial shearing against surfaces with scale heights between 0.1 and 0.3 mm and a moderate increase between 0.3 and 0.72 mm while the variation during caudal shearing is adequate. In addition, the interface friction angle reduces significantly with a longer scale length during cranial shearing and moderately during caudal shearing.
- The scale geometry ratio L/H as a function of the interface friction angle helps understand the interface anisotropy response. A small L/H ratio produces a higher interface friction angle and dilative response, whereas surfaces with higher L/H ratios tend to be contractive and have lower shear resistance.
- The difference in the interface friction angle depends on the shearing direction. Compared to caudal → cranial test case, cranial → caudal exhibited a less difference. This is due to the fact that caudal first shearing process densifies the sand around scales and as a result, cranial second shearing mobilizes a higher interface friction against the denser sand. However, cranial first shearing develops a larger individual passive wedge, and the opposite shearing direction (caudal second) follows the uniform shear bands.

## Data Availability

The data used is included in this manuscript.
